# Report of Treasurer

**Published:** 1895-07

**Authors:** 


*Report of Treasurer, F. S. Davis, M. D.*

Frank S. Davis, Treasurer, in account with the Society of Homœopathicians.

| | | |
|---|---|---|
| Dr. | | |
| To cash received for dues, . . . . . . | $80.00 | |
| " " " interest, . . . . | 1.29 | |
| | —— | $81.29 |
| Cr. | | |
| By cash paid for printing, . . . . . . | $19.25 | |
| " on hand, . . . . . . . . . . | 62.04 | |
| | —— | $81.29 |

It was moved and carried that the reports of the Secretary and Treasurer be accepted and approved.

The Committee on the Seal, Dr. Thurston, Chairman, presented a design which was accepted by the Society.

Dr. Thurston presented an amendment in writing to strike out Sections V and VI of the Declaration of Principles and the last four words of Section IV.

Dr. Biegler presented an amendment in writing to substitute "and" for "with" in the second line of Section VII of the By-Laws.

Dr. Thomson presented an amendment in writing to change "charges in writing" to "written charges" in Section XIII of By-Laws.

